# Medical and Psychiatric Comorbidities in Bipolar Disorder: Insights from National Inpatient Population-based Study

**DOI:** 10.7759/cureus.5636

**Published:** 2019-09-12

**Authors:** Sadaf Hossain, Pranita Mainali, Narmada Neerja Bhimanadham, Sundus Imran, Naveed Ahmad, Rikinkumar S Patel

**Affiliations:** 1 Psychiatry, Brookdale Hospital, Brooklyn, USA; 2 Psychiatry, Washington DC VA Medical Center, Washington DC, USA; 3 Psychiatry, Aarupadai Veedu Medical College, Puducherry, IND; 4 Neurology, Indiana University School of Medicine, Indianapolis, USA; 5 Psychiatry, University of Texas, Houston, USA; 6 Psychiatry, Griffin Memorial Hospital, Norman, USA

**Keywords:** bipolar disorder, bipolar mania, bipolar depression, comorbidities, inpatient psychiatry, nis, nationwide inpatient sample

## Abstract

Objectives

The objective of this study was to analyze the differences in the prevalence and association of medical and psychiatric comorbidities in bipolar disorder (BD) patients versus the general inpatient population.

Methods

A cross-sectional analysis was conducted using the national inpatient sample (NIS). Using the international classification of diseases, ninth revision (ICD-9) diagnostic codes, we extracted the BD inpatients and then obtained information about comorbidities. The odds ratio (OR) of comorbidities in BD inpatients were evaluated using a logistic regression model.

Results

Hypertension (31.1%), asthma (11.7%) and diabetes, obesity, and hypothyroidism (11% each) were the prevalent medical comorbidities found in BD inpatients. Hypothyroidism, asthma, and migraine were seen in BD inpatients (OR 1.59, OR 1.37 and OR 1.23; respectively) compared to general inpatients. Drug abuse (33.5%), anxiety disorders (31.8%), and alcohol abuse (18.3%) were the most prevalent psychiatric comorbidities in BD inpatients. They had a seven-fold higher likelihood of comorbid borderline personality disorders compared to general inpatients. Among other psychiatric comorbidities, the odds of the association were higher for drug abuse (OR 4.33), ADHD (OR 3.06), and PTSD (2.44).

Conclusion

A higher burden of medical and psychiatric comorbidities is seen in BD inpatients compare to the general inpatient population. A collaborative care model is required for early diagnosis and management of these comorbidities to improve the health-related quality of life.

## Introduction

Bipolar disorder (BD) is one of the most distinct forms of psychiatric illness that affects three percent of the United States (US) population. It is also one of the leading causes of disability and premature mortalities worldwide [1] due to the increased risk of suicide and medical comorbidities [2-3]. The life expectancy of patients with BD is reduced by ten years or more [4]. Although many factors may account for poor health in patients with psychiatric illness, the main reason for increased mortality and morbidity are mostly due to preventable risk factors like diabetes, obesity, smoking, and cardiovascular/metabolic conditions, many of which are related to individual lifestyle choices [5-6]. However, with proper diagnosis and treatment, individuals can lead a productive life [6].

The correlation between psychiatric and medical comorbidities is increasingly becoming a focus of healthcare providers. Past studies have shown that BD patients were significantly more likely to have medical comorbidities, including three or more chronic medical conditions (41% versus 12%) when compared with controls [6]. Advancements in technology have enabled us to understand psychiatric illnesses from a genetic perspective. Although a specific conclusion has not been reached, there have been reports of linkage of certain psychiatric illnesses with specific genes and chromosome loci [7]. Hereditary illnesses are the result of complex anomalies in gene expression, and it is not uncommon to see more than one disease emerging as a byproduct of those errors. It is possible for psychiatric and medical disorders to stem from such anomalies simultaneously and affect an individual with BD [8].

The focus of this paper was to identify if there are any increased odds of association of medical and psychiatric comorbidities in a BD versus non-BD inpatient population. Our study can seed future studies and collaborative healthcare models where bipolar patients are assessed for a spectrum of medical comorbidities to improve patient outcomes. As per our literature search and review, this will be the first nationwide inpatient study to evaluate the prevalence and association of the various comorbidities including major depressive disorder (MDD), anxiety disorders, attention-deficit/hyperactivity disorder (ADHD), borderline personality disorder (BPD), post-traumatic stress disorder (PTSD), drug abuse and alcohol abuse, diabetes, hypertension, obesity, rheumatoid arthritis, hypothyroidism, asthma, multiple sclerosis, and migraine in BD adult inpatients.

## Materials and methods

Data source

We performed a retrospective cohort analysis of the nationwide inpatient sample (NIS) database (2010 to 2014) from the healthcare cost and utilization project (HCUP) of the agency for healthcare research and quality (AHRQ) [9]. The NIS provides discharge patient data from a 20% sample of 4,400 hospitals across 45 states in the US [9], and when discharge weights [10] are applied to the data, the number of hospitalizations represent the national estimates. Diagnostic information in the NIS is detected using the international classification of diseases, ninth revision (ICD-9) codes.

Inclusion criteria and outcome variables

We included adult inpatients (age 18 and above) with a primary ICD-9 discharge diagnosis for BD (296.40, 296.41, 296.42, 296.43, 296.44, 296.50, 296.51, 296.52, 296.53, 296.54, 296.60, 296.61, 296.62, 296.63, 296.64 or 296.7).

Demographic variables studied included age (18-34, 35-50, 51-64, 65 and above), gender (male or female), and race (Caucasian, African American, Hispanic, or other) and median household income [10]. The medical and psychiatric comorbidities were based on past literature and identified using ICD-9 diagnosis codes and HCUP clinical classification software (CCS) codes [11-12].

Statistical analysis

We compared non-BD and BD inpatient cohorts using bivariate analysis to evaluate the differences in demographics and comorbidities. Multivariable logistic regression model adjusted for demographics (age, sex, and race) was used to evaluate odds ratio (OR) of medical and psychiatric comorbidities (categorical data) in BD inpatient population by comparing it with the non-BD inpatients (reference category). All statistical analyses set a priori at <0.01 were conducted on the statistical package for the social sciences (SPSS) version 25 (International Business Machines Corporation, Armonk, New York).

Ethical approval

Individual identifiers (KEY_ID) [10] were used to protect the patient identity and other clinical information. The use of NIS under the HCUP does not require approval from an institutional review board as the NIS is a publicly available de-identified database [9].

## Results

We analyzed total 27,566,280 hospitalizations, and the inpatient prevalence of BD was 1.15%. About three-fourths of the BD inpatients were constituted by young adults (18-34 years) and elders (35-50 years) and these populations had 6.4 to 6.8 times higher odds for BD related hospitalization compared to older age above 65 years. A higher proportion of BD inpatients were female (57.7%), but when compared with non-BD inpatients, the male had 1.1-fold higher odds (95% CI; 1.10 to 1.12) of BD-related hospitalization compared to female. Seventy-three percent of BD inpatients were white and comparatively other races (including Hispanic and African American) did not have higher odds of association for BD-related hospitalization.

Drug abuse (33.5%) was the most prevalent psychiatric comorbidity in BD inpatients, followed by anxiety disorder (31.8%) and alcohol abuse (18.3%). Compared to non-BD inpatients, BD inpatients had two to four times higher odds of comorbid drug and alcohol abuse, as shown in the regression model. Though borderline personality disorder (BPD) was seen in only 6.9% BD inpatients, yet they had 6.9-fold higher odds (95% CI 6.09 - 7.07) compared to non-BD inpatients. ADHD (OR = 3.055; 95% CI 2.998-3.112) when compared with the general inpatient population. The likelihood of comorbidities such as ADHD (OR 3.06, 95% CI 2.99 to 3.11), anxiety disorder (OR 2.10; 95% CI 2.08 to 2.12) and PTSD (OR 2.44; 95% CI 2.39 to 2 47) was higher in BD inpatients compared to the non-BD inpatients.

Among medical comorbidities, the most prevalent comorbidities were hypertension (31.1%), and asthma (11.7%) followed by diabetes, obesity, hypothyroidism (11% each). Among these BD inpatients had higher odds of comorbid asthma (OR 1.37, 95% CI 1.35 to 1.38) and hypothyroidism (OR 1.59, 95% CI 1.58 to 1.62). Migraine was less prevalent in BD inpatients (5.5%) but had higher odds of association (OR 1.23, 95% CI 1.21 to 1.25) compared to 2.6% non-BD inpatients. The odds of association of demographic characteristics and comorbidities in bipolar disorder inpatients are shown in Table 1.

**Table 1:** Demographic characteristics and comorbidities in bipolar disorder inpatients The proportion of non-bipolar disorder and bipolar disorder inpatients were obtained using cross-tabulation. Significant *P*-values ≤0.01 at 95% confidence interval. Odds ratio generated by the logistic regression model and adjusted for age, gender and race. MDD, major depressive disorder; ADHD, attention-deficit/hyperactivity disorder; PTSD, post-traumatic stress disorder

Variables	Non-bipolar disorder	Bipolar disorder	Odds ratio	95% Confidence interval	P-value
Total inpatients	27250255	316025	-	-	-
Age at admission, %					
18 – 34 years	19.5	36.4	6.43	6.33–6.53	< 0.001
35 – 50 years	15.6	32.8	6.81	6.71–6.91	< 0.001
51 – 64 years	22.7	22.7	3.91	3.85–3.97	< 0.001
+65 years	42.2	8.0	Reference		
Sex, %					
Male	40.9	42.3	1.11	1.10–1.12	< 0.001
Female	59.1	57.7	Reference		
Race, %					
Caucasian	68.7	72.6	Reference		
African American	14.7	14.7	0.81	0.79–0.82	< 0.001
Hispanic	10.6	8.0	0.65	0.64–0.66	< 0.001
Other	6.1	4.6	0.72	0.71–0.73	< 0.001
Psychiatric comorbidities, in %					
No comorbidities	-	-	Reference		
MDD	1.2	1.6	0.51	0.49–0.52	< 0.001
Anxiety	10.3	31.8	2.10	2.08–2.12	< 0.001
ADHD	0.5	5.2	3.06	2.99–3.11	< 0.001
Borderline personality	0.2	6.9	6.93	6.81–7.07	< 0.001
PTSD	0.8	10.1	2.44	2.39–2.47	< 0.001
Alcohol abuse	4.7	18.3	1.94	1.92–1.96	< 0.001
Drug abuse	4.7	33.5	4.33	4.29–4.37	< 0.001
Medical comorbidities, in %					
No comorbidities	-	-	Reference		
Diabetes	19.1	11.2	0.96	0.95–0.97	< 0.001
Hypertension	50.5	31.1	0.86	0.85–0.87	< 0.001
Obesity	13.4	11.1	0.82	0.81–0.83	< 0.001
Asthma	6.6	11.7	1.37	1.35–1.38	< 0.001
Rheumatoid arthritis	2.9	1.4	0.64	0.63–0.66	< 0.001
Hypothyroidism	12.0	11.0	1.59	1.58–1.62	< 0.001
Migraine	2.6	5.5	1.23	1.21–1.25	< 0.001
Multiple sclerosis	0.4	0.4	0.82	0.77–0.87	< 0.001

## Discussion

Our study showed that 36.4% of the BD inpatients were young adults (18-34 years) compared to non-BD inpatients (19.5%) in the same counterpart. This was reversed in the elderly population (age 65+) where hospitalizations due to BD were eight percent compared to non-BD inpatients (42.2%). These results correlated with findings from previous studies that stated similar prevalence of BD in the elderly i.e., 8% to 10% of geriatric psychiatric admissions [13]. The racial disparity was evident as our finding showed that the BD inpatients were constituted majorly by Caucasians (72.6%), followed by African Americans (14.7%), and Hispanics (8%) which was also seen in a recent nationwide study [11]. The reason for this imbalance was seen as people of African ancestry with BD have higher rates of misdiagnosis in comparison to people of non‐African ancestry with BD [14].

This study describes the analysis of the prevalence of the spectrum of medical and psychiatric comorbidities in BD inpatients. Hypertension was identified as the most common comorbidity among BD inpatients (31.1%) which correlates with the general global population seen in a systematic study from 2000 to 2010 (28.5% in high-income countries and 31.5% in low- and middle-income countries) and nationwide inpatient study by Patel et al. [11,15]. The global asthma report shows that about 4.3% of adults (age: 18-45 years) experienced symptoms of asthma in 2014, whereas comorbid asthma was seen in a higher proportion of BD inpatients (11.7%) in our study [16]. Females with BD have 1.75 times higher risk for asthma [11]. When we compared with the general inpatient population (non-BD cohort), the odds of association for asthma was 1.4 times higher in BD inpatients.

We found that hypothyroidism was present in eleven percent of the patients with BD which was almost three times higher than that seen in the general US population (4.6%) as per the recent report by the endocrine society [17]. The gender-wise risk for hypothyroidism was higher in females by three times [11]. When we compared with the non-BD cohort, BD inpatients have 1.6 times higher likelihood for hypothyroidism. Associations between BD and hypothyroidism have always been high whether the effect of treatment agents such as lithium on the thyroid gland is taken into consideration or not [18].

It was also noted that migraine was present in 5.5% of patients with BD which was also seen in previous studies showing strong associations predominantly in subjects with a confirmed family history of BD, suicidal attempts and childhood physical abuse [19]. In addition to the higher prevalence, migraine in patients with BD showed a more complicated disease course than patients without BD [20]. Genetic factors, inflammation, and oxidative stress have been implicated in the past as to affecting the co-occurrence of migraine and BD [21].

Patients with BD have shown vulnerability to develop metabolic disorders such as obesity and diabetes. As per the national diabetes statistics report, diabetes is seen in 14.9% of women and 15.3% of men, which is higher than that seen in the bipolar inpatients (11.2%) in our study [22]. The National Health Interview Survey (1997-2012) concluded that comorbid obesity is present in 38.3% of women and 34.3% of men, which is about three times higher than that seen in the bipolar inpatients (11.1%) in our study [23]. When we compared with the general inpatient population, diabetes, hypertension, and obesity had lower odds of association with BD inpatients. Among the BD population, women have a higher risk of obesity (by two times) and diabetes (by three times) [11].

Our study found that substance use disorders were present in an alarming 51.8% of patients with BD. When compared with general inpatients, BD inpatients have four times higher odds of drug abuse/dependence and two times for alcohol abuse/dependence. A previous study has shown that among substances, alcohol use (42%) had the highest prevalence followed by cannabis use (20%) and other illicit substance use (17%) [24]. There exist a strong association between anxiety disorders and BD [25]. Similarly, our study showed that anxiety disorder was the most common comorbid psychiatric disorder in BD patients (31.8%) with two times higher odds compared to general inpatients. BD inpatients had the highest likelihood for BPD (seven times) compared to general inpatients. Females have about 1.6 times higher risk for anxiety and BPD compared to males [11]. About half of the BD patients with comorbid BPD are young adults (18-35 years), and they are at higher needs for acute inpatient care due to suicidal risk [26].

Our study also had some limitations. First, this study was based on an administrative database, and it lacks clinical information at the patient level. NIS data on bipolar disorder is limited to hospitalizations only and do not include data from ambulatory settings. Due to the study's retrospective nature, selection bias is possible. The prevalence of comorbidities in study participants may vary when compared to that of the general population as well as other bipolar populations as our participants were chosen from the inpatient database. Despite these limitations, NIS remains an excellent population-based inpatient representation of associations of disease and comorbidities. Despite the study's retrospective nature, the chances of recall bias are likely to be minimal, given that it is an administrative database with primary and secondary diagnostic codes and other clinical information gathered at the time of hospitalization as well as discharge. The best attribute of this study is the nationally representative sample provided by the NIS dataset, as well as a uniform data collection obtained over five years through ICD-9 diagnostic codes. Another strength is its large sample size of 27,566,280 inpatients and data reliability, as the information is coded independently of the individual practitioner; this would, therefore, minimize reporting bias, as the large sample size increases the power to detect differences.

## Conclusions

A higher burden of medical and psychiatric comorbidities is seen in BD inpatients compare to the general inpatient or non-BD population. Among medical comorbidities, there is a higher risk of hypothyroidism, asthma, and migraine in BD patients. A collaborative care model is required for early diagnosis and management of these comorbidities to improve the health-related quality of life.
